# Apatinib targets both tumor and endothelial cells in hepatocellular carcinoma

**DOI:** 10.1002/cam4.1664

**Published:** 2018-08-14

**Authors:** Chaoxu Yang, Shukui Qin

**Affiliations:** ^1^ Post‐Doctoral Research Center in Nanjing General Hospital of Eastern Theater Command Nanjing China; ^2^ Cancer Center of BaYi Hospital Affiliated to Nanjing University of Traditional Chinese Medicine Nanjing China

**Keywords:** anti‐angiogenesis, apatinib, hepatocellular carcinoma, molecular biology, VEGFR‐2

## Abstract

Hepatocellular carcinoma (HCC) is one of the most commonly diagnosed malignancies worldwide with poor prognosis and tends to be hypervascular. Aberrant expression of the vascular endothelial growth factor 2 (VEGFR‐2) has been implicated in the progression of HCC and represents a valid target for anticancer therapy. Apatinib, a small molecule inhibitor of VEGFR‐2 tyrosine kinase, shows strong antitumor activity in various tumors. This study is designed to evaluate the activity of apatinib on both human umbilical vein vascular endothelial cells (HUVECs) and HCC cell lines (in vitro and in vivo), and also to investigate the characteristics and possible mechanisms underlying these effects by molecular biology methods. Following the results in our study, apatinib inhibited phosphorylation of VEGFR‐2 in HUVECs and blocked in vitro endothelial cell migration and tube formation. Concentration‐dependent antiproliferative effects of apatinib were also observed in all 6 HCC cell lines including SK‐Hep‐1, HepG2, Hep3B, Huh‐7, PLC/PRF/5, SMMC‐7721. Moreover, response to apatinib of HCC cell lines was significantly correlated with VEGFR‐2 expression level. Additionally, apatinib significantly inhibit VEGF‐triggered VEGFR‐2 phosphorylation and activation of downstream signaling molecules such as Akt and ERK1/2 in HCCs. Apatinib can also induce a cell cycle arrest at G2/M phase and promote HCC apoptosis tested in vitro. In vivo data showed that apatinib can effectively inhibit tumor growth, decreased angiogenesis, as well as induced HCC apoptosis (in some tumors), and thus prolonged animal survival in a mouse xenograft model of human HCC. Our findings suggested that apatinib is a highly potent, oral active anti‐angiogenic, and anti‐HCC agent. The results from current study provide a clear biological rationale to evaluate apatinib as a new agent in HCC in clinical setting, especially for the VEGFR‐2 overexpression ones.

## 1 INTRODUCTION

Liver cancer is one of the most frequently occurring malignancies worldwide, with an estimated 0.78 million new cases each year, ranking fifth in males and ninth in females during 2012.^1^ Moreover, liver cancer is the second leading cause of cancer‐related mortality, with an estimated 0.75 million deaths globally, only behind lung cancer.^1^ Hepatocellular carcinoma (HCC) accounts for 70% to 90% of all liver cancer cases.^2^ Notably, about 75%‐80% of HCC cases reported each year occurs in Asian countries, particularly, China alone accounts for more than 50% of HCC cases and deaths worldwide.^3^, ^4^ The leading risk factor for HCC is hepatitis B virus (HBV) infection, and other relevant risk factors include aflatoxin exposure, heavy alcohol drinking, tobacco smoking, overweight, and diabetes.^5^, ^6^, ^7^

Surgical resection, liver transplantation, and local ablation are potentially curative treatments for HCC patients, with 5‐year survival rates of 60%‐80% in resection and 40%‐70% in ablation.^8^, ^9^ But only 15%‐20% patients, who are diagnosed with early‐stage HCC, are available for these curative treatments.^10^ Additionally, nearly 70% of patients develop a recurrence within 5 years after surgical resection or ablation, which compromises overall survival in these patients.^8^, ^9^ Therefore, it is essential to explore novel treatment strategies against HCC.

Hepatocellular carcinoma is a typical hyper‐vascular tumor characterized by a florid intra‐tumoral vasculature.^11^ Angiogenesis has been shown to play a critical role in the development and progression of HCC, which provides a theoretical evidence for antiangiogenic strategies as therapy.^11^ Vascular endothelial growth factor (VEGF) is a powerful pro‐angiogenic factor involved in hepatocarcinogenesis, neovascularization, invasiveness, and metastatic potential of HCC.^12^, ^13^ The overexpression of VEGF in tumor tissues can be used as a prognostic factor in patients with HCC after treatment.^14^ Current data demonstrated that VEGF/VEGFR‐2 mediated signaling cascades are involved in proliferation, migration, survival, and permeability changes of vascular endothelial cells.^15^ Thus, anti‐angiogenesis via blockade of VEGF/VEGFR‐2 pathway provides attractive targets for the development of new anti‐HCC drugs.

Apatinib is a small‐molecular tyrosine kinase inhibitor that selectively binds and potently suppresses VEGFR‐2 and then blocks VEGFR‐2‐mediated angiogenesis.^16^ As one of the latest orally antiangiogenic agents, apatinib shows encouraging preclinical and clinical results in the treatment of various solid tumors.^16^, ^17^, ^18^, ^19^, ^20^, ^21^ It has been approved for advanced gastric cancer after second‐line chemotherapy by China Food and Drug Administration.^17^ In addition, we have launched a phase II clinical trial of apatinib in advanced HCC. Results suggested that apatinib monotherapy is effective and safe in advanced HCC.^22^ However, the underlying mechanism of apatinib against HCC remains unclear. This study is designed to evaluate the effects of apatinib on human umbilical vein vascular endothelial cells (HUVECs) and HCC cell lines both in vivo and in vitro, as well as to investigate the characteristics and possible mechanisms underlying these effects. The results are expected to provide experimental data and theoretical basis for the anti‐HCC clinical practice.

## 2 MATERIALS AND METHODS

### 2.1 Reagents

Apatinib was obtained from Jiangsu Hengrui Medicine Co., Ltd. For in vitro studies, apatinib was dissolved in 100% dimethyl sulfoxide and diluted to the desired concentration with Dulbecco's modified Eagle's medium (DMEM). For in vivo studies, apatinib was diluted in 0.5% w/v of carboxymethyl cellulose and 0.5% w/v of glucose solution.

### 2.2 Cell lines

Human umbilical vein vascular endothelial cells were purchased from the American Type Culture Collection. HCC cell lines including SK‐Hep‐1, HepG2, Hep3B, Huh‐7, PLC/PRF/5, SMMC‐7721, and human hepatic cell line L02 were obtained from Cell Bank of Type Culture Collection of Chinese Academy of Sciences (Shanghai, China). HUVECs were maintained in Dulbecco's modified Eagle's medium (DMEM)/F12 containing 20% fetal bovine serum (FBS) supplemented with 100 μ/mL penicillin, 0.1 mg/mL streptomycin and 50 μ/mL gentamicin. HCC cells and L02 cells were cultured in DMEM containing 10% FBS supplemented with 100 μ/mL penicillin, 0.1 mg/mL streptomycin, and 50 μ/mL gentamicin. All cells were kept in a humidified chamber at 37°C containing 5% CO_2_.

### 2.3 Cell proliferation assay

The viability of HCC cells under treatment of apatinib at different concentration was measured using the Cell Counting Kit‐8 (CCK‐8, Dojindo Molecular Technologies, Gaithersburg, MD, USA). Briefly, cells grown to logarithmic phase were passaged and placed into a 96‐well plate at a density of 5 × 10^3 ^cells/well. Then, apatinib at a gradient concentration was added to the cells. After 48 hours of incubation, the mixture was treated with CCK‐8 reagent and incubated for another 1 hour at 37°C. The cell viability was determined by measuring the absorbance at 540 nm in a microplate reader. Each experiment was repeated four independent times.

### 2.4 Cell migration and tube formation assay

Changes in HUVEC migration in vitro after apatinib treatment were detected using Transwell and scratch wound‐healing assay. For Transwell assay, HUVEC migration was assessed with a modified Boyden chamber which contains an 8‐μm polycarbonate membrane filter coated with gelatin. Firstly, the Matrigel was mixed with serum‐free DMEM at a volume ratio of 1:5 and blocked with 1% fatty acid‐free bovine serum albumin (BSA) in phosphate‐buffered saline (PBS). Then, the upper surface of the filter was filled with 2.5 mg/mL Matrigel at 37°C for 4‐5 hours. Afterward, cells at 1.5 × 10^5^/mL were seeded onto the upper chamber and the lower chamber was added with DMEM containing 20% FBS. The chamber was incubated at 37°C for 20‐24 hours. Nonmigrated cells were scrapped from the upper surface of the membrane with a cotton swab. Cells migrating to the lower chamber were stained with 0.1% crystal violet after immobilized by glutaraldehyde and counted in random 10 fields under inverted microscope. For scratch wound‐healing assay, cells grown to logarithmic phase were seeded to a 6‐well plate (5 × 10^5 ^cells/well) overnight. A scratch was made in the cell layer with a sterile p200 pipette tip. The plates were washed with PBS for three times to remove the cell debris and added with serum‐free DMEM for incubation. Photographs were taken every 6 hours under microscope, and cell migration was analyzed using Image J software.

To visualize the microvessels, HUVECs at density of 1 × 10^4 ^cells/well were seeded onto the Ibidi μ‐Slide (IBIDI, Munich, Germany) coated with Matrigel and treated with apatinib at different concentration. Then, cells were incubated at 37°C and 5% CO_2_. At 6 hours postseeding, cells were observed under a phase‐contrast microscope and photographed at 200× magnification. Results were analyzed using WimTube Quantitative Tube Formation Image Analysis online software.

### 2.5 Western blot

Western blot analysis was performed on frozen tissues and cultured cells. Tissues or cells were homogenized in lysis buffer (5 mmol/L DTT, 5 mg/mL aprotinin, 0.5 mmol/L phenylmethylsulfonyl fluoride, and 5 mg/mL leupeptin in 10 mmol/L Tris buffer) on ice for 10‐20 minutes and ultrasonically dispersed. Then, mixture was separated by centrifugation at 18750g and the supernatant was collected. Total protein was quantified using a bicinchoninic acid (BCA) kit. After SDS‐PAGE, isolated proteins were transferred onto nitrocellulose membranes. After blocked in 5% BSA for 1 hour, membranes were incubated with primary monoclonal antibodies against target at 4°C overnight. On the next day, membranes were incubated in appropriate horseradish peroxidase‐conjugated secondary antibodies at 37°C for 1 hour after washing with Tris‐buffered saline containing 0.1% Tween‐20 thrice. GAPDH or β‐actin was used as an internal control. The proteins were visualized using an ECL detection system (Santa Cruz Biotechnology Inc., Santa Cruz, CA, USA), and the protein bands were quantified using the Quantity‐One software (Bio‐Rad, Hercules, CA, USA).

### 2.6 Flow cytometry

The effects of apatinib on cell cycle and apoptosis were detected by a flow cytometer. HCCs grown to logarithmic phase were seeded in a 25 cm^2^ flask at a density of 1 × 10^6^ cells/flask. After 24 hours, apatinib was added to the cultured cells and the mixture was incubated for continuous 36 hours. Cells were collected every 12 hours, trysinized and fixed in 1 mL 80% cold ethanol at 4°C for 15 minutes. Then cells were centrifugated at 200g for 5 minutes, and the cell pellets were resuspended in 500 μL propidium iodine (PI, 10 μg/mL) containing 50 μL/mL RNase (Sigma, St Louis, MO, USA). Then, cells were incubated at 37°C for 30 minutes in the dark and filtered through nylon mesh (53 μm). Cell cycle distribution was calculated using MODFIT LT 2.0 software from 10 000 cells (Becton Dickinson, San Diego, CA, USA).

For cell apoptosis, cells were washed with incubation buffer (25 mmol/L HEPES/NaOH, 140 mmol/L NaCl and 5 mmol/L CaCl_2_). After centrifugation at 88g for 5 minutes, cells were labeled with FITC‐Annexin V (1 μg/mL) and PI (1 μg/mL) according to the manufacturer's introduction. The mixture was incubated at room temperature in the dark for 10‐15 minutes, followed by the addition of SA‐Flous buffer at 4°C. Then, the mixture was incubated for another 20 minutes at 4°C. Cell apoptosis was detected by flow cytometry: FITC‐/PI‐ cells were defined as viable, FITC+/PI‐ cells were defined as early apoptotic, FITC+/PI+ cells were defined as necrotic.

### 2.7 Animals and treatment

BALB/cA‐specific pathogen‐free female nude mice, weighing 16‐20 g, were purchased from Sino‐British SIPPR/BK Lab Animal Co., Ltd. (Shanghai China) and kept in pathogen‐free cages provided with standard rodent chow and sterile water ad libitum. After acclimatization, cells at 1 × 10^7^/mL were inoculated subcutaneously into the back of mice. Tumors were established and allowed to grow 150‐200 mm^3^. Then, animals were randomly divided into four groups (eight animals for each group) and given different treatments: (a) control; (b) apatinib (50 mg/kg); (c) apatinib (100 mg/kg); and (d) apatinib (200 mg/kg). Drugs were intragastrically administered to mice for consecutive 14 days and rested for 7 days. To detect the effects of apatinib on tumor volume and weight in mice, the length (*a*) and the width (*b*) of tumors were measured every 2 days using a caliper and tumor volume was calculated by the following formula: tumor volume = 0.52 × *a *× *b* ^2^. Twenty‐one days after apatinib administration, mice were sacrificed by cervical dislocation, and tumors were removed and weighed.

Additionally, to detect the effects of apatinib on mouse survival, apatinib was given intragastrically to mice, once daily, for 14 days followed by 7‐day off period in each 21‐day cycle. Mouse deaths and their survival time were recorded during treatment period.

### 2.8 Immunohistochemistry

After a cycle (21 days) of apatinib administration, mice were sacrificed by cervical dislocation. Tumor tissues were collected, fixed in 4% formaldehyde, and embedded in paraffin. Then samples were cut into consecutive 4‐μm sections and stored at ‐80°C for further analyses.

For immunohistochemical analysis, paraffin sections were deparaffinized in xylene and hydrated in alcohol (100%‐95%‐85%‐75%). Then, sections were blocked in 3% H_2_O_2_ for endogenous peroxidase activity and underwent antigen retrieval in 0.01 mol/L citrate buffer. After washing with PBS, sections were blocked in 1% BSA and incubated with mouse primary antibody directed against CD31, an endothelial cell marker at 4°C overnight. Then sections were incubated with secondary goat anti‐mouse antibodies (DAKO) at 37°C for 45 minutes. After washing with PBS, sections were stained with DAB and counterstained in hematoxylin. Microvessel density (MVD) measurement was performed as previously described.^23^

Apoptosis was detected using DeadEnd^™^ Fluorometric TUNEL System (Promega, Madison, WI, USA) as described preciously,^24^ and the apoptotic cells was detected by a Nikon fluorescence microscopy.

### 2.9 Statistical analysis

All statistical analyses were performed using SPSS 19.0 software. Data were presented as mean ± standard deviation. Comparisons among multiple groups were performed using one‐way analysis of variance followed by Fisher's least significant difference test, and between‐groups were performed by independent *t* test. An association between two numeric variables was evaluated by calculating Pearson's correlation coefficient. Kaplan‐Meier method was used to estimate survival curves. *P *<* *0.05 was considered statistically significant.

## 3 RESULTS

### 3.1 Inhibitory effects of apatinib on HUVECs

**Figure 1:**
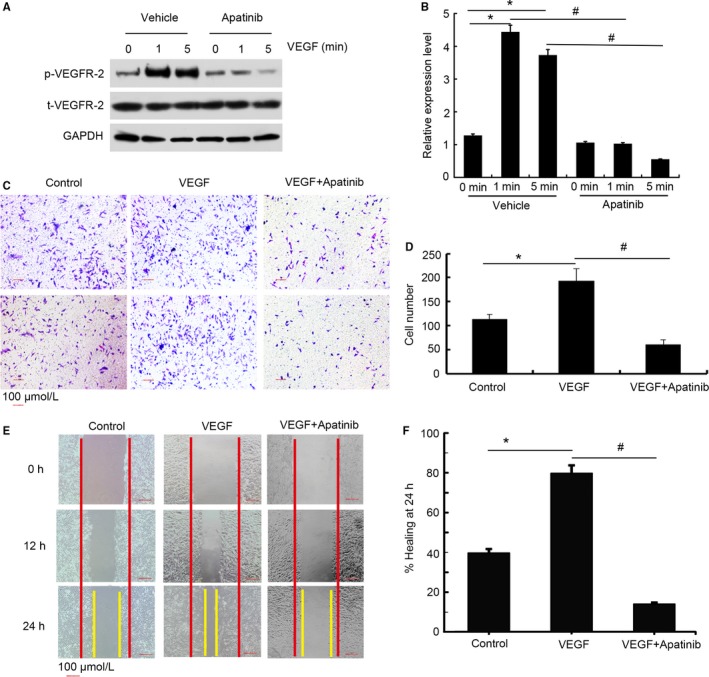
Apatinib Blocks VEGF‐Induced VEGFR‐2 Phosphorylation in HUVECs and Inhibits HUVEC Migration. A, HUVECs were treated with 20 nmol/L apatinib or vehicle. VEGF at final concentration of 30 ng/mL was then added into HUVECs. At 0, 1, and 5 min after addition of VEGF, HUVECs were subjected to Western blot analysis. GAPDH was used as an internal control. B, Quantification of Western blot data. *P <* *0.05 compared to HUVECs at 0 min after VEGF addition, ^#^ *P *<* *0.05 compared to HUVECs treated with vehicle. C and E, HUVECs were treated with vehicle, VEGF (30 ng/mL) or VEGF (30 ng/mL) + Apatinib (0.5 μmol/L) and subjected to Transwell (C) or scratch wound healing assay (E). D and F, Quantification of Transwell assay data (D) and wound healing assay data (F). *P <* *0.05 compared to HUVECs treated with vehicle, ^#^ *P *<* *0.05 compared to HUVECs treated with VEGF

We first tested the effects of apatinib on VEGF stimulated VEGFR‐2 tyrosine phosphorylation in HUVECs. The incubated HUVECs were treated with 20 nmol/L apatinib or vehicle. VEGF at final concentration of 30 ng/mL was added into HUVECs that were treated with apatinib or not. At 0, 1, and 5 minutes after addition of VEGF, cells were collected and total cellular protein extracts were subjected to Western blot analysis. In HUVECs without apatinib treatment, addition of VEGF at 1 and 5 minutes significantly increased the content of phosphorylated VEGFR‐2 (*P *<* *0.05), while the content of total VEGFR‐2 changed indistinctly during whole treatment process (Figure 1A,B). However, the content of phosphorylated VEGFR‐2 was markedly reduced in apatinib‐treated HUVECs at 1 and 5 minutes after addition of VEGF (Figure 1A,B) compared to the HUVECs treated with vehicle (*P *<* *0.05). These results suggested that apatinib can inhibit VEGF‐triggered VEGFR‐2 phosphorylation in HUVECs.

Next, we tested the effects of apatinib on HUVECs migration by both Transwell and scratch wound healing assays. HUVECs were harvested and divided into follow groups: vehicle (without VEGF and apatinib), VEGF (30 ng/mL), and VEGF (30 ng/mL) + Apatinib (0.5 μmol/L). Then, these HUVECs were subjected to Transwell and scratch wound healing assays. The results were displayed in Figure 1C‐F. In Transwell assay, VEGF induction led to greater migration of HUVECs compared to the cells in control group (*P *<* *0.05), while addition of apatinib significantly inhibited VEGF‐induced HUVECs migration (*P *<* *0.05). In vitro scratch wound healing assay also suggested that VEGF markedly enhanced wound closure when HUVECs were exposed to VEGF at either 12 or 24 hours after scratch. However, HUVECs treated with VEGF plus apatinib exhibited significantly lower degrees of wound closure compared to those treated with VEGF alone, as seen in monolayers photographed at 24 hours after wound incision and quantified as closure speed (*P *<* *0.05).

**Figure 2:**
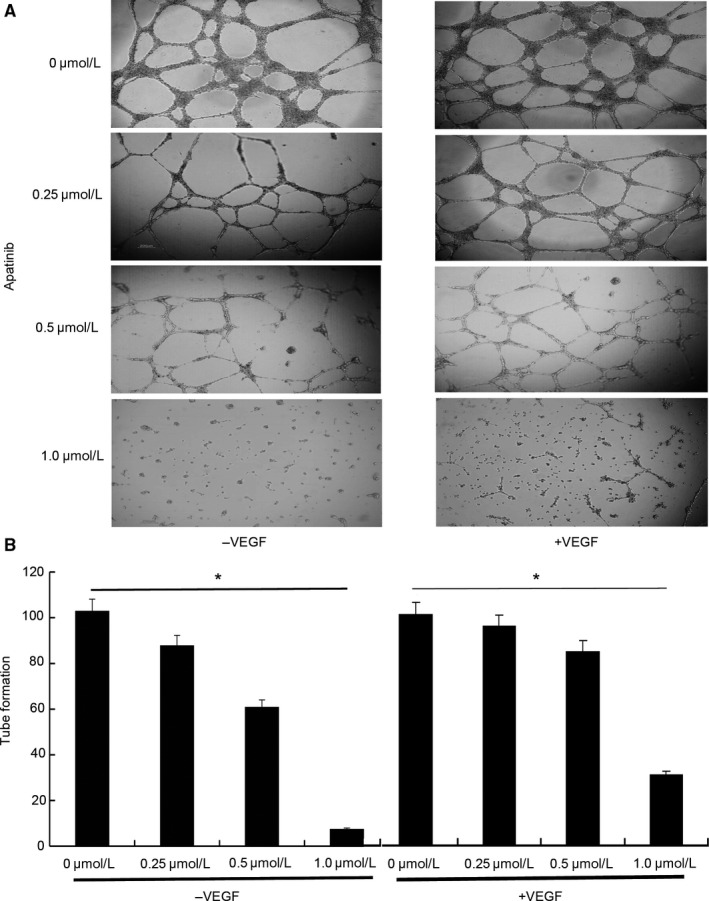
Apatinib Inhibits Tube Formation of HUVECs. A, HUVECs were seeded on the Matrigel surface and treated with apatinib at different concentration (0, 0.25, 0.5 and 1.0 μmol/L) in the presence of VEGF (30 ng/mL) or not. At 6 h postseeding, cells were observed for microvessels formation under a phase‐contrast microscope and photographed at 200 ×  magnification. B, WimTube Quantitative Tube Formation Image Analysis online software was used to quantify the capillary‐like tube formation in HUVECs in the absence and presence of VEGF (30 ng/mL) after apatinib treatment at 0, 0.25, 0.5, and 1.0 μmol/L. *P <* *0.05 compared to HUVECs treated with vehicle

The development of capillary tubes and sprouting of new capillaries are hallmarks of angiogenesis during solid tumor growth. To evaluate the effects of apatinib on this reorganization stage during angiogenesis, tube formation assay was performed. Briefly, HUVECs were seeded on the surface of Matrigel and treated with apatinib at different concentration (0, 0.25, 0.5 and 1.0 μmol/L). As shown in Figure 2A, human umbilical endothelial tube formation was obviously inhibited by apatinib, whether VEGF (30 ng/mL) was present or not, and the inhibitory effects were dose independent. The WimTube Angiogenesis Analysis platform was used to quantify the capillary‐like tube formation in HUVECs in the absence and presence of VEGF (30 ng/mL) after apatinib treatment at different concentration. Following the results in Figure 2B, apatinib at 1.0 μmol/L significantly inhibited human umbilical endothelial tube formation in HUVECs compared to that in HUVECs without apatinib treatment with or without VEGF (*P *<* *0.05).

### 3.2 Apatinib inhibits cell proliferation of HCCs, which is correlated with VEGF

**Figure 3:**
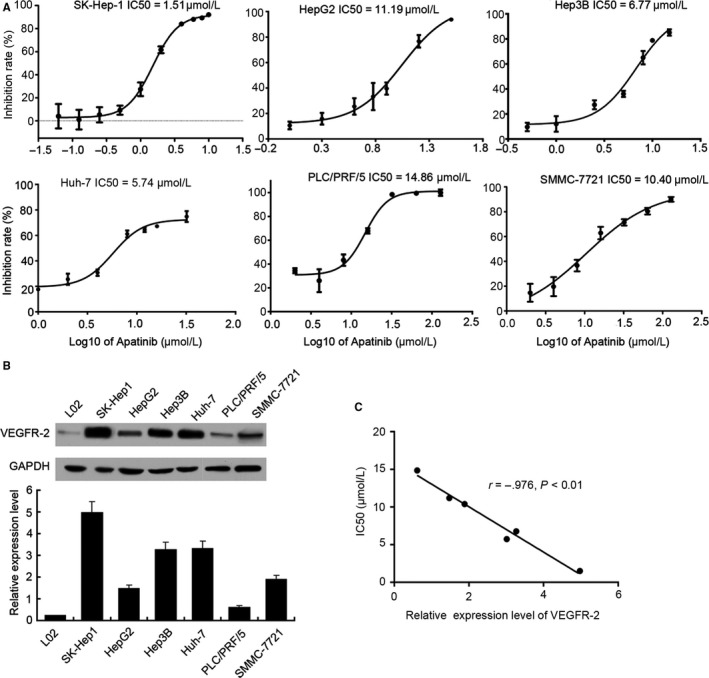
Apatinib Inhibits HCC Cell Proliferation, Which is Correlated with VEGF Expression in HCC. A, A panel of six commonly used HCC cell lines including SK‐Hep‐1, HepG2, Hep3B, Huh‐7, PLC/PRF/5, and SMMC‐7721 grown to logarithmic phase were placed into 96‐well plates at a density of 5 × 10^3 ^cells/well and treated with apatinib at gradient concentration. After 48 h of incubation, cell viability was detected by CCK‐8 kit. B, The expression of VEGFR‐2 in 6 HCC cell lines and human hepatic L02 cell was detected and quantified by Western blot. C, Pearson's correlation coefficient was calculated to measure the correlation between IC50 value of apatinib in 6 HCC cell lines and VEGFR‐2 expression in these cells

Besides HUVECs, we also detected the effects of apatinib on HCCs. Six commonly used HCC cell lines including SK‐Hep‐1, HepG2, Hep3B, Huh‐7, PLC/PRF/5, and SMMC‐7721 were treated with increasing concentrations of apatinib for 48 hours, and the cell viability was determined by CCK‐8 assay. The results suggested that apatinib suppressed the proliferation of all six common HCC cell lines in a dose‐independent manner. But the IC50 values varied widely between the individual cell lines (Figure 3A). The IC50 value ranged from 1.51 μmol/L in SK‐Hep‐1 cells to 14.86 μmol/L in PLC/PRF/5 cells among the tested six cell lines.

In addition, Western blot was performed to test and quantitate the expression of VEGFR‐2 in 6 HCC cell lines and normal human hepatic L02 cell. Based on the results, VEGFR‐2 was upregulated in all 6 HCC cell lines compared to the normal human hepatic L02 cell, while VEGFR‐2 expression level was also different from each other. The highest VEGFR‐2 expression level was detected in SK‐Hep‐1 cells that presented the smallest IC50 value, while the lowest was found in PLC/PRF/5 cells that presented the largest IC50 value (Figure 3B) among 6 HCC cell lines.

Following above results, VEGFR‐2 overexpression may associate with a consistently higher sensitivity of apatinib treatment across 6 HCC cell lines tested in vitro. In addition, a direct and liner inverse correlation exists between sensitivity to apatinib and VEGFR‐2 expression after comparing the IC50 value of apatinib with the relative expression levels of VEGFR‐2 in the 6 HCC cell lines tested (Figure 3C, *r* = −0.976, *P *<* *0.01). Furthermore, considering the possible difference of mechanisms due to the difference in VEGFR‐2 expression levels, SK‐Hep‐1 and PLC/PRF/5 cells were chosen for further analyses.

### 3.3 Apatinib induces HCC cell cycle arrest and apoptosis, as well as blocks VEGF mediated downstream activation

**Figure 4:**
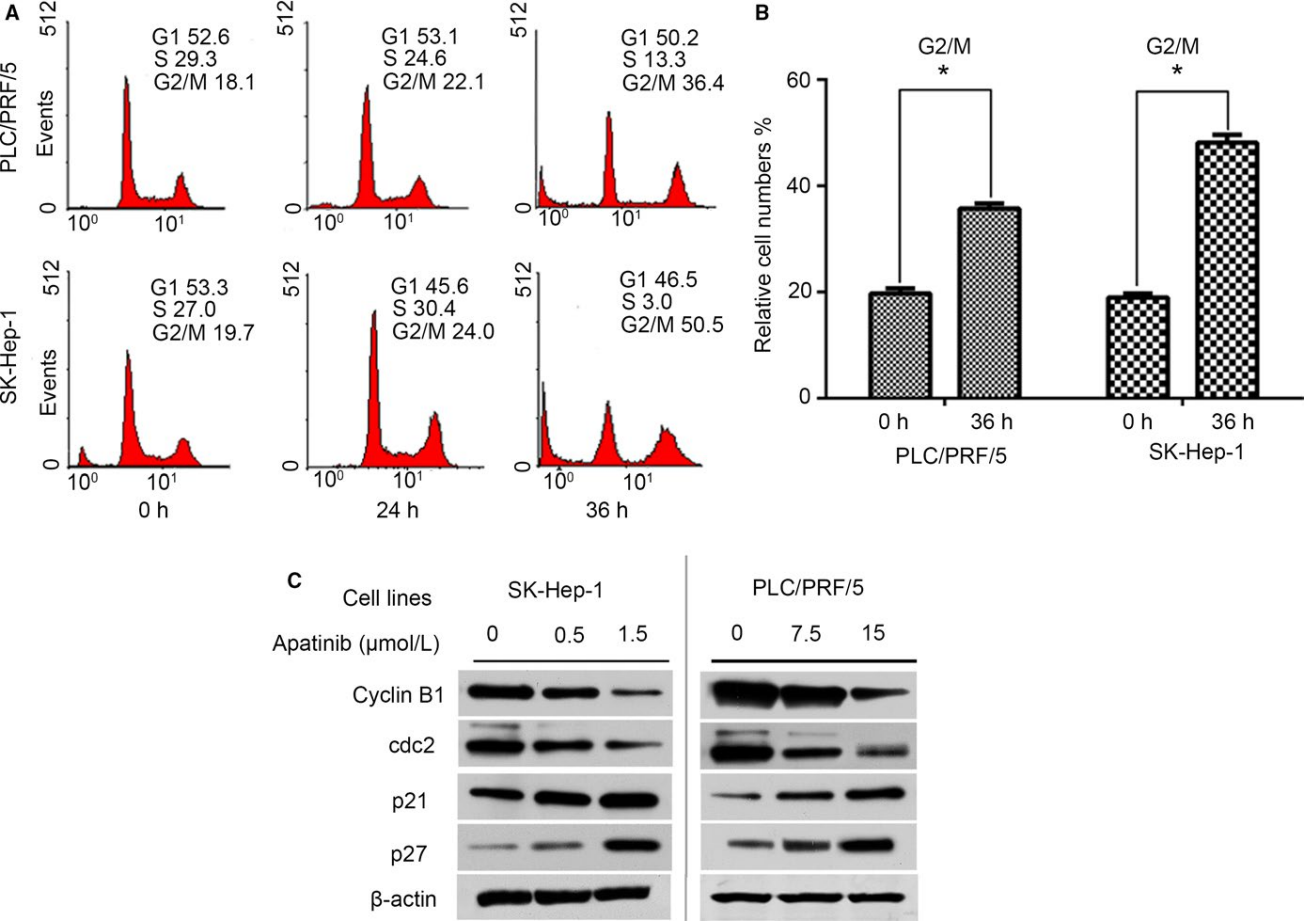
Apatinib Induces G2/M Phase Arrest in SK‐Hep1 and PLC/PRF/5 Cells. A, Cells were treated with apatinib (1.5 μmol/L for SK‐Hep1 cell and 15 μmol/L for PLC/PRF/5 cells) and analyzed for cell cycle by flow cytometry at 0, 24, and 36 h after drug treatment. B, Quantification of cells arrested in G2/M phase at 0 and 36 h after apatinib treatment. *P <* *0.05 compared to the relative number of cells in G2/M phase at 0 h after apatinib treatment. C, Cells were treated with apatinib at 0, 0.5 and 1.5 μmol/L for SK‐Hep‐1 cells and 0, 7.5 and 15 μmol/L for PLC/PRF/5 cells. After 36 h of incubation, cells were lysed and processed for western blot analysis to detect the expression of cell cycle‐associated proteins including cyclin B1, cdc2, p21, and p27. Β‐actin was used as an internal control

Flow cytometry was performed to detect the effects of apatinib on HCC cell cycle and apoptosis. As shown in Figure 4A, apatinib at IC50 concentration (1.5 μmol/L for SK‐Hep1 cell and 15 μmol/L for PLC/PRF/5 cells) induced a G2/M‐phase cell cycle arrest as early as 24 hours, and after 36 hours of apatinib treatment, 36.4% PLC/PRF/5 cells and 50.5% SK‐Hep‐1 cells were arrested in G2/M phase. Figure 4B presented that treatment of apatinib at 36 hours induced a significant increase in G2/M‐phase cells compared to those at the beginning of apatinib treatment (*P *<* *0.05). The following Western blot analysis confirmed these results. The cyclinB1/cell division cycle 2 (cdc2) complex plays a key role in promoting the G2/M phase transition. Additionally, the members of the cyclin‐dependent kinase (CDK) inhibitor family of protein, such as p21 and p27, regulate G1/S and G2/M phase transitions by inactivating the cyclin/CDK complexes. The expression of cyclinB1 and cdc2 was markedly decreased after apatinib treatment, while the expression levels of p21 and p27 were significantly higher in apatinib‐treated cells compared to the controls (Figure 4C).

**Figure 5:**
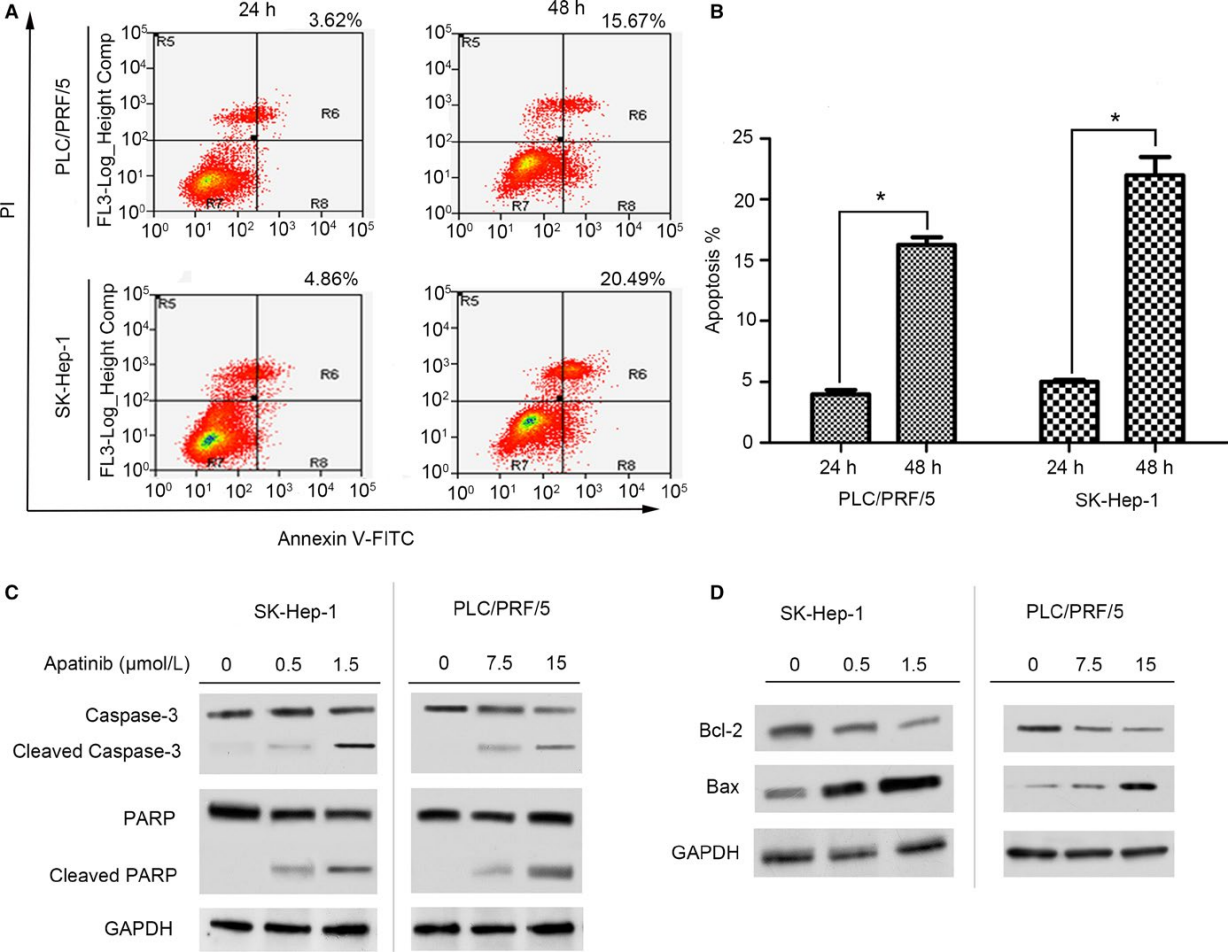
Apatinib Induces Cell Apoptosis in SK‐Hep1 and PLC/PRF/5 Cells. A, Cells were treated with apatinib (2.0 μmol/L for SK‐Hep1 cell and 15 μmol/L for PLC/PRF/5 cells). After collected, cells were stained with FITC‐labeled annexin V and PI and analyzed for apoptosis by flow cytometry at 24 and 48 h after apatinib treatment. B, Quantification of apoptotic cells at 24 and 48 h after apatinib treatment. *P <* *0.05 compared to apoptosis rate at 24 h after apatinib treatment. C, Cells were treated with apatinib at 0, 0.5, and 1.5 μmol/L for SK‐Hep‐1 cells and 0, 7.5 and 15 μmol/L for PLC/PRF/5 cells. After 48 h of incubation, cells were lysed and processed for Western blot analysis to detect the expression of cell apoptosis associated proteins including caspase‐3, cleaved caspase‐3, PARP, and cleaved PARP. GAPDH was used as an internal control. D, Cells were treated with apatinib at 0, 0.5, and 1.5 μmol/L for SK‐Hep‐1 cells and 0, 7.5 and 15 μmol/L for PLC/PRF/5 cells. After 48 h of incubation, cells were lysed and processed for Western blot analysis to detect the expression of bio‐markers of mitochondrial‐dependent apoptosis including Bcl‐2 and Bax. GAPDH was used as an internal control

Besides inducing G2/M phase arrest, apatinib also promotes HCC apoptosis in a time‐dependent manner. Following the results of flow cytometry analysis (Figure 5A), there were 3.62% apoptotic PLC/PRF/5 Cells and 4.86% apoptotic SK‐Hep‐1 cells at 24 hours after apatinib treatment, which increased to 15.67% and 20.49%, respectively, at 48 hours after apatinib treatment. The counts of apoptotic PLC/PRF/5 cells and SK‐Hep‐1 cells were significantly higher at 48 hours than those at 24 hours of apatinib treatment (Figure 5B). In addition, apatinib treatment increased the expression of apoptosis‐related protein including cleaved‐caspase3 and poly ADP‐ribose polymerase (PARP), while downregulated the expression of anti‐apoptotic protein Bcl‐2 and upregulated the expression of pro‐apoptotic protein Bax, supporting the results that apatinib (around IC50 concentration) induced HCC apoptosis through the mitochondrial‐dependent pathway in vitro (Figure 5C‐D).

**Figure 6:**
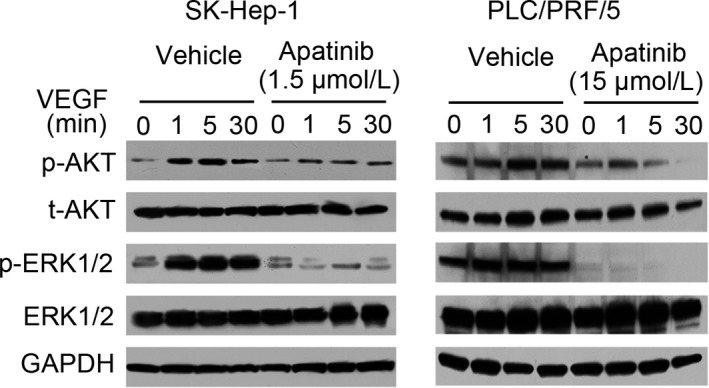
Apatinib blocks VEGF/VEGFR mediated downstream activation in SK‐Hep1 and PLC/PRF/5 cells. Cells were collected and treated with vehicle or apatinib (1.5 μmol/L for SK‐Hep1 cell and 15 μmol/L for PLC/PRF/5 Cells). After 6 h of incubation, cells were processed for Western blot analysis

The activation of VEGF/VEGFR‐2 signaling induces the phosphorylation of various downstream signal transduction mediators, including protein kinase B (AKT) and mitogen‐activated protein kinase (MAPK), which play a central role in tumor angiogenesis by promoting endothelial cell proliferation, migration, and tube formation in HCC. To investigate the molecular mechanisms responsible for the biological effects of apatinib on HCC cells, the phosphorylation of ERK1/2 and Akt was determined by Western blot. Incubated SK‐Hep‐1 and PLC/PRF/5 cells were collected and treated with vehicle or apatinib at IC50 concentration, respectively. Then, cells were incubated at 37°C and 5% CO_2_ for 6 hours, lysed and processed for Western blot analysis. As observed in Figure 6, apatinib treatment at IC50 concentration obviously inhibited the activation of Akt and ERK1/2 in both SK‐Hep‐1 cells and PLC/PRF/5 cells.

### 3.4 Apatinib inhibited tumor growth in HCC xenograft mice

**Figure 7:**
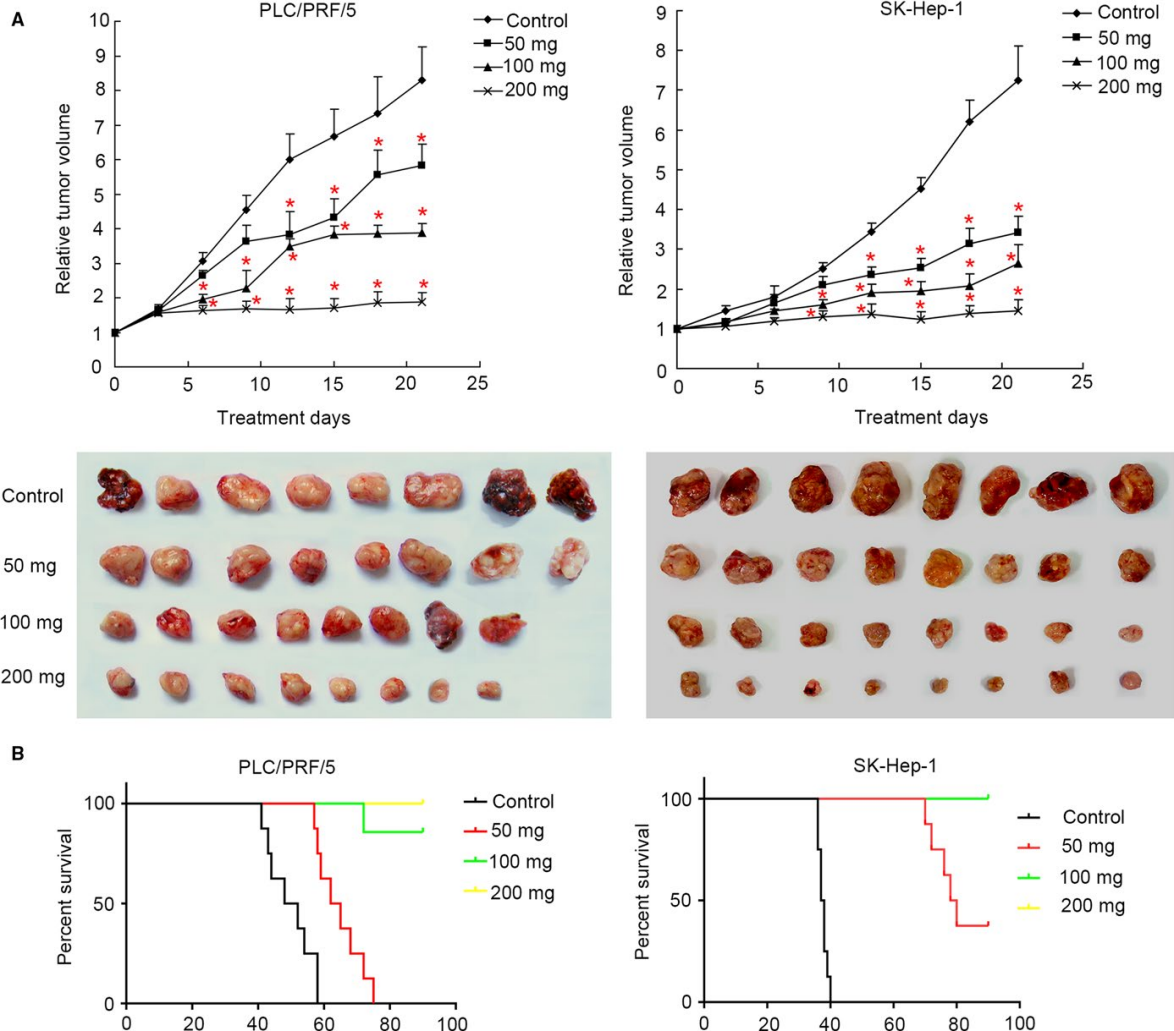
Apatinib Inhibits Tumor Growth and Prolongs Survival of HCC Xenograft Mice. A, BALB/cA nude mice were subcutaneously inoculated with SK‐Hep1 or PLC/PRF/5 cells. When tumor grew to 150‐200 mm^3^, animals were randomly divided into four groups (eight animals for each group) and given different treatments (0, 50, 100 and 200 mg/kg apatinib). Then tumor volume in mice was analyzed every 2 d. *P <* *0.05 compared to control. B, Kaplan‐Meier survival curves were plotted in both PLC/PRF/5 and SK‐Hep‐1 xenograft mice after apatinib treatment at different concentration (0, 50, 100, and 200 mg/kg apatinib)

We also evaluated the antitumor potential of apatinib in HCC xenograft mouse model in vivo. As displayed in Figure 7A, oral administration of apatinib once daily at 50 mg/kg for 14 days, 100 and 200 mg/kg for 6 days significantly inhibited both PLC/PRF/5 and SK‐Hep‐1 tumor growth compared to the control (*P *<* *0.05). And the maximal efficacy on tumor growth inhibition was achieved at the dose of 200 mg/kg of apatinib. No deaths were observed in all mice during 21‐day observation time. Mice weight among four groups differed indistinctly in both PLC/PRF/5 and SK‐Hep‐1 bearing mice (data not shown).

Additionally, we also explored the effects of apatinib on tumor‐bearing mice survival, and the results are shown in Figure 7B. Apatinib at 50, 100, and 200 mg/kg could significantly prolong the survival of both PLC/PRF/5 (median survival, control vs 50, 100, 200 mg/kg: 50 vs 63.5 days, not estimated, not estimated, *P *<* *0.05) and SK‐Hep‐1 xenograft mice (median survival, control vs 50, 100, 200 mg/kg: 37.5 vs 79 days, not estimated, not estimated, *P *<* *0.05) compared to the control.

### 3.5 Antitumor activity of apatinib in vivo

**Figure 8:**
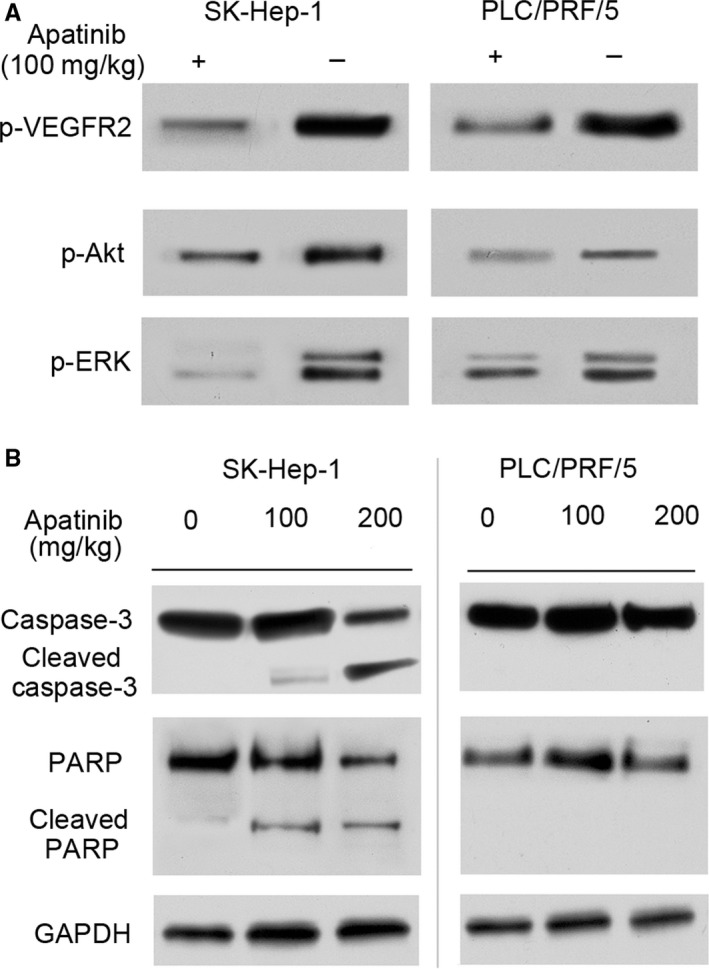
Apatinib Inhibits the Expression of VEGF/VEGFR Downstream Signaling Protein and Cell Apoptosis‐related Protein in vivo. A, Tumor tissues were harvested from mice treated with apatinib at 0 or 100 mg/kg. Western blot was performed on the frozen tumor tissues to detect the expression of phosphorylated VEGFR‐2, Akt and ERK. B, Tumor tissues were harvested from mice treated with apatinib at 0, 100, or 200 mg/kg. Then, Western blot was performed to detect the expression of apoptosis associated protein including caspase‐3, cleaved caspse‐3, PARP, and cleaved PARP in tumor tissues

**Figure 9:**
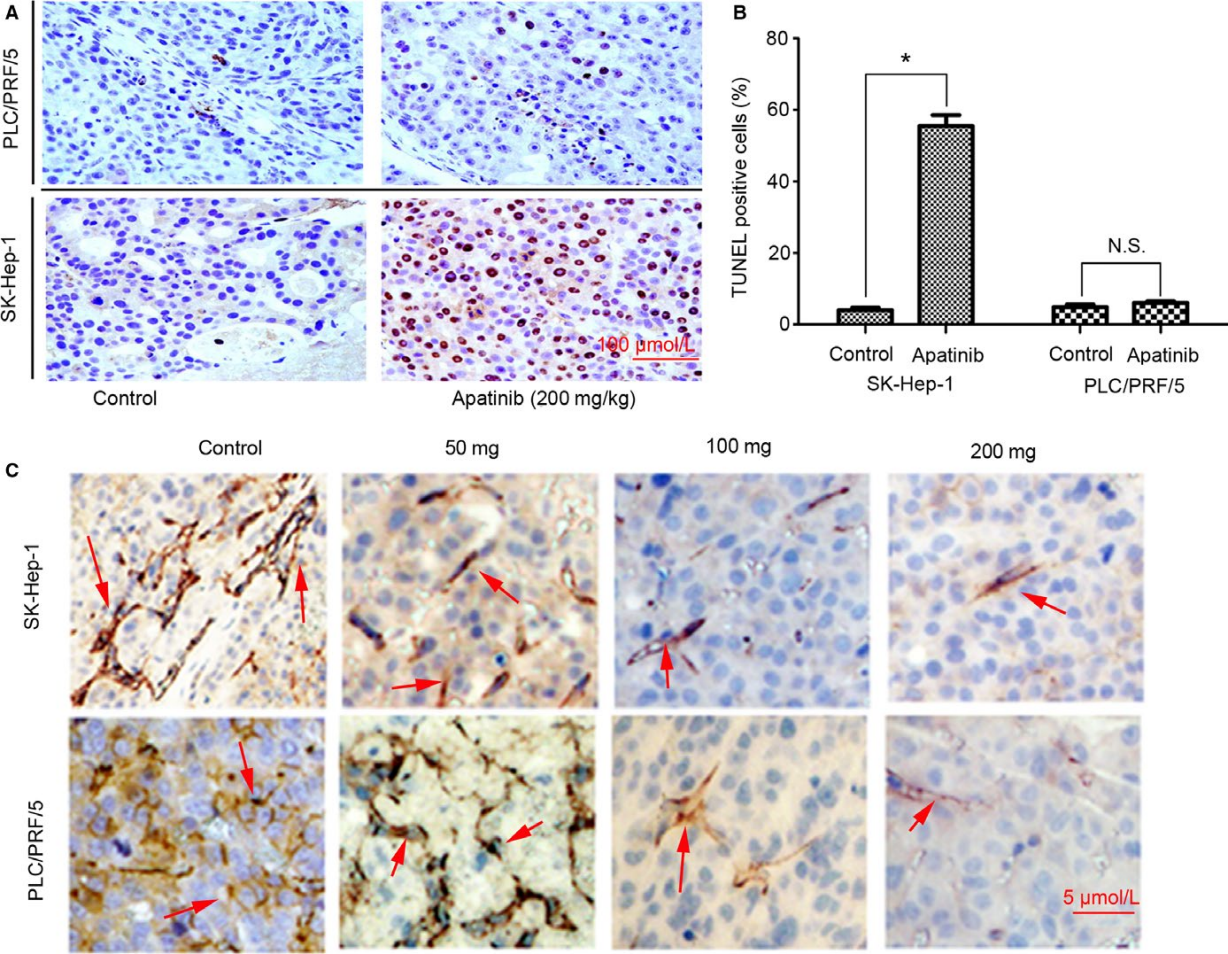
Apatinib Induces Apoptosis and Inhibits Microvessel Formation in HCC in vivo. A, TUNEL analysis was performed on xenograft tumor specimens to detect cell apoptosis in apatinib (0 or 200 mg/kg) treated mice (SK‐Hep‐1 and PLC/PRF/5 xenograft mice). B, Quantification of TUNEL‐positive staining cells on xenograft tumor specimens in apatinib (0 or 200 mg/kg)‐treated mice (SK‐Hep‐1 and PLC/PRF/5 xenograft mice). *P <* *0.05 compared to control; N.S. not significant compared to control. C, Microvessel densities in tumors after apatinib treatment at different concentration (0, 50, 100, and 200 mg) that were detected by IHC staining of CD31

To further examine target modulation in vivo, tumors were harvested, homogenized, and analyzed by Western blot for downstream proteins expression of drug targets. Consistent with the in vitro Western blot results, quantification of p‐VEGFR‐2, p‐Akt, p‐ERK revealed almost 50% decrease in apatinib (100 mg/kg)‐treated tumors (Figure 8A). Interestingly, the cleaved caspase‐3 and cleaved PARP can only be found in the SK‐HEP‐1 tumor treated with 100 or 200 mg/kg apatinib, not in PLC/PRF/5 tumor with or without apatinib treatment (Figure 8B). The TUNEL assay on Xenograft tumor specimens also showed a marked apoptosis can only be observed after apatinib treatment (200 mg/kg) in the SK‐HEP‐1 tumor. But for PLC/PRF/5 tumor, apatinib at treated dose seemed not to induce tumor apoptosis (Figure 9A,B). However, the following IHC staining of CD31 showed the microvessel densities of both tumors (SK‐HEP‐1 and PLC/PRF/5 tumor) in the treatment group were obviously reduced compared to the control group (Figure 9C).

## 4 DISCUSSION

As Judah Folkman first proposed the hypothesis that tumor growth depends on angiogenesis in 1971,^25^ inhibition of angiogenesis has become an important therapeutic strategy in the treatment of various tumors. HCC is a typical hypervascular tumor, with high levels of VEGF receptor expression in tumor tissues.^26^, ^27^ Tumor angiogenesis plays an important role in the occurrence, development, and metastasis of HCC.^28^ It is also closely correlated with tumor stage and prognosis.^29^ A number of studies have been carried out to investigate the agents that targets VEGF axis for advanced HCC. Sorafenib, a multityrosine kinase inhibitor that targets both angiogenic and tumorigenic receptors, is the first and only agent that has been approved by regulators globally as a standard therapy in patients with advanced HCC due to an overall survival benefit compare to the placebo.^30^ Although the introduction of sorafenib in advanced HCC changed the clinical landscape for this malignancy, sorafenib has some limitations such as at best modest, transient benefits, and toxicity challenges.^31^ As such, it is necessary to continuingly explore more effective therapies to combat this fatal disease.

Apatinib is a small‐molecular VEGFR TKI that highly binds and inhibits VEGFR‐2.^16^ By blocking VEGF axis, apatinib effectively inhibits the proliferation, migration, and tube formation of HUVEC.^16^ A phase II clinical trial of apatinib in advanced HCC presented the effectiveness and safety of apatinib monotherapy in advanced HCC.^22^ The present study was performed to explore the antitumor activity of apatinib in HCC both in vitro and in vivo, aiming to provide evidence support of apatinib in the treatment of HCC in clinical practice.

Similar to previous description, apatinib exerted a strong inhibitory effect on VEGFR‐2 phosphorylation, HUVEC migration, tube formation in this study.^16^ These results verified the anti‐angiogenesis activity of apatinib. Moreover, besides HUVECs, VEGFs, and their receptors are found to be expressed in many tumor cells. VEGF/VEGFR signaling is required in tumor cells to stimulate their proliferation and invasive growth in an autocrine and angiogenesis‐dependent manner.^32^ Recent studies have reported the possible molecular mechanisms involved in the direct anticancer action of apatinib in various cancer cell lines.^33^, ^34^, ^35^ These findings suggested that apatinib may also have the potential to directly suppress HCC cell growth. As shown in the present study, the expression of VEGFR‐2 was markedly higher in 6 HCC cell lines compared to normal controls. Apatinib showed direct inhibitory effects on HCC cell lines, and the IC50 value ranged from 1.51 to 14.86 μmol/L. Moreover, the IC50 value was negatively correlated with VEGFR‐2 expression in different HCC cells, indicating that the efficacy of apatinib does differ widely between individual cell lines that expressed different levels of VEGFR‐2. The relationship between the antiproliferative effects of apatinib and VEGFR‐2 expression has been indicated in gastric cancer lines.^36^ The results in our study suggested that the expression of VEGFR‐2 could be used to evaluate apatinib sensitivity in patients with HCC, providing experimental evidence for individualized treatment of HCC and population screening of apatinib‐sensitive HCC.

The mechanism of inhibitory activity of apatinib in HCC cells was further investigated by detection of cell cycle changes, cell apoptosis, as well as the expression of potential downstream signaling pathways. Based on our results, the inhibitory effects of apatinib on HCC growth were involved in many aspects: inducing cell cycle arrest at G2/M phase via blockade of cyclin B1/cdc2 complex and upregulation of p21 and p27; inducing cell apoptosis via activation of caspase‐3 and PARP cleavage; and inhibiting VEGF‐induced Akt and ERK phosphorylation.

In a xenograft model of HCC, apatinib could significantly delay the tumor growth in mice. The in vivo antitumor effects of apatinib in HCC was slightly different for the tested cell lines with different levels of VEGFR‐2 expression. For the PLC/PRF/5 xenograft HCC which possessed relatively lower expression level of VEGFR‐2, the antitumor effects of apatinib could be attributed to blockade of cell cycle progression, reduction in neovascularization, and inhibition of VEGFR‐2/Akt/ERK pathway activation. For the SK‐HEP‐1 cells with the relatively higher VEGFR‐2 expression level, besides the mechanisms mentioned above, the antitumor effects of apatinib in HCC mice could be attributed to induction of tumor cell apoptosis through the mitochondrial dependent pathway. These results suggested that the pharmacologically relevant dose of apatinib on inducing cell apoptosis was not same for different HCC cell lines with different VEGFR‐2 expression. The current data showed that apatinib was more potent in inhibiting HCC tumor with high expression of VEGFR‐2. This finding was important as recent progress in molecular‐targeted anticancer therapies highlighted the need for identification of suitable biomarkers for treatment efficacy. Following our results, the expression level of VEGFR‐2 could be used to evaluate the sensitivity of apatinib treatment in HCC. Certainly, this result should be confirmed in further prospective clinical trials.

## 5 CONCLUSION

In summary, apatinib is a highly potent VEGFR‐2 inhibitor and target both endothelial and HCC cell in vitro and in vivo. Our findings provide a rationale for clinical trials of apatinib as a single agent or in combination with other agents in patients with HCC, especially those with overexpressed VEGFR‐2, which warrant further investigation in clinical practice.

## CONFLICT OF INTEREST

All authors declared no conflict of interest.
